# Comprehensive analysis of gene expressions in ileal mucosa of chickens fed paddy rice and their IgA response to oral vaccination

**DOI:** 10.1016/j.psj.2025.104823

**Published:** 2025-01-15

**Authors:** M. Yamamoto, A. Ueno, H. Watanabe, M. Okamoto, K. Furukawa, A. Murai

**Affiliations:** Laboratory of Animal Nutrition, Department of Animal Science, Graduate School of Bioagricultural Sciences, Nagoya University, Nagoya, Japan

**Keywords:** Paddy rice, IgA, Oral vaccination, Short-chain fatty acid, Chicken

## Abstract

Paddy rice ingestion increases intestinal mucin secretion and production by enhancing *MUC2* gene expression and epithelial turnover. In this study, we performed a comprehensive analysis of intestinal gene expression in chickens fed paddy rice and investigated whether the intestinal IgA response was modified by paddy rice ingestion. Furthermore, we investigated the possible involvement of gut fermentation. Layer male chicks were divided into two groups according to diet i.e., corn or paddy rice at 650 g/kg diet, which were given for 14 consecutive days at 7 d of age. The ileal gene expression levels in both groups were compared using DNA microarray analysis. A total of 120 genes were upregulated >1.5-fold in the paddy rice group, whereas 159 genes were downregulated <1.5-fold. Remarkably, the gene expression levels of immunoglobulin heavy chain α (*IGHA*), immunoglobulin J chain (*IGJ*), and immunoglobulin light chain λ chain region (*IGLL1*), which constitute immunoglobulin A, decreased 3–10 times in the paddy rice group. Infectious bursal disease virus (IBDV) vaccine were orally administered to chickens fed corn or paddy rice to examine the intestinal immune response. Paddy rice ingestion diminished both the total IgA concentration and IBDV-specific IgA in the bile. Cecal total short-chain fatty acid and butyric acid concentrations decreased by 30 % in the paddy rice group compared to those in the corn group. In conclusion, feeding paddy rice to chickens decreased intestinal IgA production, which was partly attributable to the low fermentability of paddy rice in the intestinal tract.

## Introduction

Whole-grain and ground paddy rice are valuable grain sources in poultry diets (Nanto et al., 2012; Sittiya et al., 2014, 2016). Paddy rice mainly consists of the three components, husk, bran and endosperm. The primary component of the endosperm is carbohydrates, particularly starch (Yin et al., 2023). The rice bran includes various nutrients, including proteins, lipids, vitamins, minerals and fibers, and it is a notable source of vitamin E, including tocopherols and tocotrienols (Kodape et al., 2025). The γ-oryzanol, ferulic acid with anti-oxidative and anti-inflammatory properties, is also rich in the rice bran (Kodape et al., 2025). Rice hull is a complex lignocellulosic material rich in water-insoluble dietary fiber (IDF); they consist of lignin 15.4 %, cellulose 35.6 %, hemicellulose 12.0 % and ash rich in hydrated silica 18.7 % by wet weight (Friedman, 2013). The hull is 20–30 % of the whole rice grain; hence, ingestion of paddy rice could broadly affect gastrointestinal physiology, mainly through bulk forming capacity of IDF in gut contents. On the other hand, the rice hull is not easily fermented and has little nutritional value.

Our previous study indicated that paddy rice ingestion stimulated small intestinal mucin secretion and production (jejunum < ileum) in chickens more significantly than corn, brown rice, or polished rice ingestion because of its IDF-bulk-forming properties (Murai et al., 2018). MUC2 is the major secretory mucin in the intestine and forms a protective gel mucosal barrier that prevents potential pathogens and antigens from accessing the underlying epithelium (McGuckin et al., 2011). In support of this, ingestion of paddy rice protected “leaky gut” experimentally-induced by dextran sodium sulfate, whereas ingestion of corn did not (Murai et al., 2018). It has also been reported that the administration of rice hull-IDF attenuates colitis inflammation by enhancing mucin secretion and intestinal tight connectivity in mice (Tian et al., 2022). These results suggest that paddy rice ingestion can enhance intestinal barrier function through mucosal barrier development. On the other hand, feeding paddy rice to chicks suppressed the growth of *Campylobacter* in the gastrointestinal tract of broiler chicks by promoting the grinding activity of the gizzard (Nishii et al., 2016). Thus, paddy rice ingestion can also enhance intestinal barrier function by increasing gastrointestinal motility.

The increased gut viscosity caused by IDF causes the retention of bulkier and more viscous digesta along the small intestinal tract, leading to increased intraluminal pressure. Increased intraluminal pressure and stretching force affect epithelial cell turnover or the differentiation of epithelial stem cells, leading to increased numbers of goblet cells (Ito et al., 2009; Murai et al., 2018). Data regarding the effect of IDF on nutrient digestibility in chickens are limited; however, dietary IDF affects the digestibility of nutrients, either beneficially or detrimentally, depending on the dietary content (Röhe and Zentek, 2021). In addition, dietary IDF affects the bacterial metabolite profiles owing to its low fermentability. Relatively high concentrations (10 %) of dietary IDF did not affect cecal microbial composition but reduced the cecal concentration of short-chain fatty acids (SCFAs) and ammonia in broilers (Röhe et al., 2020a) and hens (Röhe et al., 2020b). Thus, paddy rice ingestion potentially affects gastrointestinal physiology and function, including digestion/absorption of nutrients and gut barrier functions, such as mucosal immunity.

We conducted a comprehensive analysis of gene expression in the small intestine using a DNA microarray to obtain insights into the physiological modifications in the small intestine of chickens fed paddy rice. We also investigated whether the intestinal IgA response was modified by paddy rice ingestion and further examined contribution of intestinal fermentation products to the IgA response.

## Materials and methods

### Animals

Fertilized eggs from commercial White Leghorn chickens were purchased from a local supplier (Julia Light, Japan Layer, Gifu, Japan). The fertilized eggs were incubated at 37 °C with a relative humidity of 58 %–68 %, turned once per hour until 18 d of incubation, and then moved to a wire-bottom hatching box. After allowing 24 h for hatching, male chicks were moved to stainless-steel cages and housed. The birds were provided with free access to water and a semi-purified diet based on isolated soybean protein extract and corn starch (Murai et al., 2018). The photoperiod was set at 16 L:8 D with lights on at 08:00 beginning at 3 d of age. Room temperature was controlled at 32 °C at 0–3 d of age, 30 °C at 3–7 d of age, 28 °C at 7–14 d of age, 26 °C at 14–21 d of age, 24 ± 2 °C after 21 d of age. The total number of experimental animals used were 50 birds (18, 16 and 16 birds in Experiment 1, 2 and 3, respectively). Animal care complied with the applicable guidelines of the Nagoya University Policy on Animal Care and Use (approval no. 2016022608, 2017030222).

Corn and paddy rice (Momiroman strain, cultivated in Japan) were analyzed according to AOAC 2006 for dry matter (DM, 934.01), crude protein (CP, 984.13, using Kjeldahl), crude fat (920.39, using ether extraction), crude fiber (978.10), and ash (942.05). The values for corn were 85.6 % DM, 6.94 % CP, 3.22 % crude fat, 1.84 % crude fiber, 1.07 % crude ash, and 72.53 % nitrogen-free extract (NFE), while that for paddy rice were 83.84 % DM, 6.48 % CP, 1.46 % crude fat, 5.81 % crude fiber, 2.75 % crude ash, and 67.34 % NFE.

### Experimental design

***Experiment 1.*** The 1-wk-old chickens were allocated into two groups (nine birds each) based on body weight (BW) to obtain a similar mean BW (60–75 g at 1 wk of age) across all groups, and each bird was caged individually. Birds were allowed free access to experimental diets containing corn or paddy rice 650 g/kg for 2 wk (Table 1). The experimental diets were formulated to meet or exceed the nutrient requirements of chickens (National Research Council, 1994), but the differences in CP and ME values among the diets were not unified. Corn and paddy rice were ground into particles <3 mm in diameter. The birds were euthanized using decapitation at 3 wk of age. The small intestine was excised, and the luminal contents were removed by flushing with PBS. Segments (50–70 mg) of the jejunum (above the Meckel's diverticulum) and ileum (adjacent to the tip of the cecum) were excised for DNA microarray and real-time gene expression analyses. These tissues were rapidly frozen in liquid nitrogen and stored at −80 °C.

**Table 1:** Composition (g/kg) of experimental diets.

Ingredients	Corn	Paddy rice
Corn	650	-
Paddy rice	-	650
Soybean protein extract	210.7	210.7
Vitamin mixture^1^	10	10
Mineral mixture^2^	58.3	58.3
Choline chloride	2	2
Soybean oil	60	60
DL-Methionine	6	6
L-Lysine	1	1
L-Threonine	1	1
L-Tryptophan	1	1
Total	1000	1000
**Calculated composition**		
Crude protein (%)	24.3	22.8
ME (kcal/kg)	3489	3090

1 Vitamin mixture provided the following (per kg of diet): nicotinic acid, 30 mg; pantothenate, 15 mg; pyridoxine, 6 mg; thiamin, 5 mg; riboflavin, 6 mg; folic acid, 2 mg; vitamin K, 750 μg; d-biotin, 200 μg; vitamin B_12_, 25 μg; vitamin A, 4000 IU; vitamin D_3_, 1000 IU; vitamin E, 75 IU.

2 Mineral mixture provided the following (per kg of diet): CaHPO_4_•2H_2_O, 20.7 g; KH_2_PO_4_, 10 g; CaCO_3_, 14.8 g; KCl, 3 g; NaCl, 6 g; MgSO_4_, 3 g; FeSO_4_•7H_2_O, 0.5 g; MnSO_4_•5H_2_O, 0.35 g; KI, 2.6 mg; CuSO_4_•5H_2_O, 40 mg; ZnO, 62 mg; Na_2_MoO_4_•2H_2_O, 8.3 mg; NaSeO_3_, 0.4 mg; CoCl_2_•6H_2_O, 1.7 mg. These amounts of vitamins and minerals satisfy the nutrient requirements of chickens.

***Experiment 2.*** The 1-wk-old chickens were allocated to two groups (eight birds each), and each bird was caged individually. The same dietary treatment as in Experiment 1 was applied to birds until 11 wk of age to eliminate maternally derived anti-infectious bursal disease virus (IBDV) antibodies. Food and water were withdrawn overnight for oral vaccination at 9 wk (61 d) of age. All birds were orally intubated with 1 mL of live IBD vaccine solution (Nobilis Gumboro D78.1000; MSD Animal Health, Japan) in the following morning, followed by the administration of 50 mL of a 10-fold diluted vaccine solution. Food was withdrawn overnight at 11 wk (75 d) of age. Blood samples were collected from the wing vein, and the birds were euthanized using decapitation. Bile was collected after laparotomy and stored at −30 °C.

***Experiment 3.*** The 1-wk-old chickens were allocated to two groups (eight birds each), and each bird was caged individually. The same dietary treatments as those used in Experiments 1 and 2 were applied for 10 wk (Table 1) and the birds were euthanized by decapitation. Cecal contents were collected to measure the SCFA concentration.

### Comprehensive gene expression analysis by DNA microarray

Total RNA was extracted from the ileum using the TRI reagent (Molecular Research Center, Cincinnati, OH, USA). The total RNA from 3 birds per treatment was pooled for each chip, and the mixed RNA (100 µg) was cleaned up and purified using an RNeasy Mini Kit. GeneChip Chicken Genome Array (Affymetrix) was used for comprehensive gene expression analysis of the purified RNA. Raw data were normalized using the MAS5 algorithm in Affymetrix GeneChip Operating Software ver. 1.4. The microarray data have been deposited in the NCBI Gene Expression Omnibus (GSE277733). Gene expression levels that increased more or decreased less than 1.5-fold in the paddy rice group compared to that in the corn group (control) were extracted as differentially expressed genes between the two groups.

### Gene expression analysis using real-time PCR

Total RNA was isolated from the ileum and jejunum and treated with DNase using the Turbo DNA-free kit (Ambion, Thermo Fisher Scientific, Waltham, MA, USA). First-strand cDNA was synthesized from the total RNA using a high-capacity cDNA Reverse Transcription Kit (Applied Biosystems). Gene expression was quantified using real-time PCR and SYBR Green (Toyobo, Osaka, Japan) according to the manufacturer's instructions. Each 15 μL qPCR mixture comprised 7.5 μL SYBR Green Master Mix, 0.45 μL each of primer (300 nM final), 1 μL of synthesized cDNA or its diluted solution (1–100-fold), and 5.6 μL PCR-grade water. The cycling parameters were initial denaturation at 95 °C for 2 min, followed by 40 cycles of denaturation at 95 °C for 15 s and primer annealing at 60 °C for 60 s, with a final cycle of 95 °C for 15 s, 60 °C for 30 s, and 95 °C for 15 s for analysis of the primer-dissociation curve. The expression data were normalized to endogenous 18S rRNA expression levels. Specific primer pairs were designed by Primer Express Software version 3.0 (Applied Biosystems) based on the analyzed sequence data or NCBI GenBank data (Table 2).

**Table 2:** Primer sequences, corresponding accession numbers, and amplification sizes.

Gene	Accession no.	Primer sequence 5′→3′	Product size (bp)
18SrRNA	AF173612	F: TCCCCTCCCGTTACTTGGAT	60
		R: GCGCTCGTCGGCATGTA	
MUC2	XM_421035	F: TTCATGATGCCTGCTCTTGTG	93
		R: CTGAGCCTTGGTACATTCTTGT	
CUBN	XM_001235155.4	F:AAGGGACGAATTGTCACAGTGA	61
		R:TGCAGTCCCCTGGGTCAT	
CYP4B1	XM_004936774.2	F:CAGCACACAGGCATTCACATG	60
		R:CAATGCAGTTCCTGGATCCA	
APOA4	XM_204938.1	F:CACTGTGCTCTGGAGGTACTTCA	99
		R:AGGGTGTTGAGCTGCTTGGT	
GAL10	NM_001001609.1	F:AGGCTTTCACTGGGCCATT	58
		R:AGCTCCTCAAGGCAGTGGAA	
IGHA	S40610	F:TGCGCCAAAGGTGCTAGTG	73
		R:GGCCCCATGCGTCGAT	
IGJ	NM_204263.1	F:TGCGTAACGGTGACCTCAAA	62
		R:TCCAGGACTTCTTCCTCAGGATT	
IGLL1	NM_001278545.1	F:GGTGTGCCTGATAAACGACTTCT	60
		R:CCATCGATCACCCAATCCA	
CALB1	NM_205513.1	F:AGCCTTGGTCTTGGCATGTT	60
		R:TTTTTGTGCCTTTCTTCAGCAA	
SLC16A1	NM_001006323.1	F:AGCAGCATCCTGGTGAACAAG	59
		R:AGGCACCCACCCACGAT	
SLC5A8	NM_416173.5	F:CCCAATCCGTTGCAAAGG	60
		R:CCATGTGAAGGTCCCACCTATAA	

### ELISA assay for immunoglobulin levels

IgG and IgA concentrations in the serum and bile were measured using a Chicken IgG and IgA ELISA Quantitation Kit (Bethyl Laboratories, Montgomery, TX, USA), according to the manufacturer's instructions. A 10,000-fold dilution of serum and a 5,000-fold dilution of bile were used to measure the IgG concentrations. Serum was diluted 4,000-fold, and bile was diluted 5,000-fold or 10,000-fold to measure IgA concentrations.

### Determination of IBDV-specific antibody titer by ELISA

The IBDV-specific antibody titer was measured using an IBD ELISA kit (IDEXX Laboratories, Westbrook, Maine, USA) according to the manufacturer's instructions. Serum and bile were diluted 500-fold and 50-fold, respectively, using a serum dilution buffer to measure the IgG titer. After the reaction, absorbance was measured at 650 nm. Serum and bile were diluted 50-fold to measure the IgA titer. HRP-conjugated IgA detection antibody (goat anti-Chicken IgA HRP conjugated, Bethy Laboratories) at a 5,000-fold dilution was used for detection.

### Measurement of SCFA concentrations in cecal contents

SCFA concentrations in the cecal contents were measured by the Institute of Nutrition and Pathology, Inc. (Kyoto, Japan). SCFA concentrations were measured using ion-exclusion HPLC, as described by Tsukahara et al. (2014), with a slight modification. Briefly, three cecal samples (0.3 g each) were randomly selected from the two groups and mixed with 0.6 mL of distilled water. Diluents were mixed with 90 μL 12 % perchloric acid (v/v). After centrifugation (15,000 × g at 4 °C for 10 min), the supernatants were filtered through a 0.45 μm cellulose acetate membrane filter (Cosmonice Filter W, Nakalai Tesque, Kyoto, Japan) and degassed under vacuum. The supernatants (5 μL) were injected into an SIL-10 autoinjector (Shimadzu, Kyoto, Japan). SCFA were separated using two serial organic acid columns (7.8 mm x 30 cm x 2 sets, Waters, Tokyo, Japan) at 45 °C with isocratic elution (0.8 mL/min) of 5 mmol/L ρ-toluene sulfonic acid aqueous solution using a solvent delivery pump (LC-10ADvp; Shimadzu). SCFA were detected with an electronic conductivity detector (Waters 431; Waters) after post-column dissociation (0.8 mL/min) with 5 mmol/L ρ-toluene sulfonic acid, 20 mmol/L bis-Tris and 100 μmol/L EDTA using a solvent delivery pump (LC-10ADvp; Shimadzu). The SCFAs were quantified using a system controller (CBM-20A; Shimadzu).

### Statistical analysis

In Experiments 1 and 2, the data were analyzed using Student's *t*-test, and in Experiment 3, the data were analyzed using the Steel–Dwass test. Statistical analysis was performed with the Excel-statistics 2010 for Windows (Social Survey Research Information, Tokyo, Japan).

## Results

### Experiment 1: microarray analysis for screening of the differentially expressed genes

There were no significant differences in final body weight and feed intake between the control group and the paddy rice group. Ileal *MUC2* gene expression (10 birds in each group) was measured using real-time PCR to select RNA samples for DNA microarray analysis. Three samples with the averaged ileal *MUC2* gene expression values were selected (1.00 ± 0.048 in the control vs. 1.56 ± 0.11 in the paddy rice, *P* < 0.01). Three selected samples were screened for differentially expressed genes. Compared to the control group, 120 genes (23 of which were unknown) from 133 probe sets were upregulated >1.5-fold in the paddy rice group. In addition, 159 genes (32 of which were unknown) from the 172 probe sets were downregulated <0.67-fold in the paddy rice group (Table 3). Among these genes, we focused on those involved in the immune system, intestinal barrier function, and other biological defenses, as well as in the utilization and absorption of nutrients (Table 4): *CUBN, CYP4B1 GAL10, CALB1, IGLL1, APOA4, SLC16A1*, and *SLC5A8*.

**Table 3:** The number of probes and genes with higher (> 1.5) or lower (0.67 <) gene expressions of ileum in paddy rice group relative to corn group.

n	Number of probes	Number of genes
GeneChip® Chicken Genome Array	37,703	32,773
Paddy rice / Corn > 1.5	133	120
Paddy rice / Corn < 0.67	172	159

**Table 4:** Notable genes upregulated > 1.5-fold or down-regulated < 0.67 in paddy rice group relative to corn group.

Gene Symbol	Gene name	Fold change	Entrez Gene
Upregulated			
*CUBN*	cubilin (intrinsic factor-cobalamin receptor)	2.46	420523
*CYP4B1*	cytochrome P450, family 4, subfamily B, polypeptide 1	2.46	424618
Downregulated			
*IGJ*	immunoglobulin J polypeptide, linker protein for immunoglobulin alpha and mu polypeptides	0.13	374117
*IGLL1*	immunoglobulin lambda-like polypeptide 1	0.15	416928
*CALB1*	calbindin 1, 28kDa	0.35	396519
*GAL10*	Gal 10	0.35	414341
*APOA4*	apolipoprotein A-IV	0.46	395780
*SLC16A1*	solute carrier family 16, member 1 (monocarboxylic acid transporter 1)	0.62	419875
*SLC5A8*	solute carrier family 5 (iodide transporter), member 8	0.54	417932

Chickens were given corn- or paddy rice-based diets from 7 to 21 days of age. Tissues were collected at 21 days, and their ileal mRNA gene expressions were analyzed by DNA microarray.

Fold change was calculated by the gene expression level in paddy rice group relative to corn group.

**Fig. 1:**
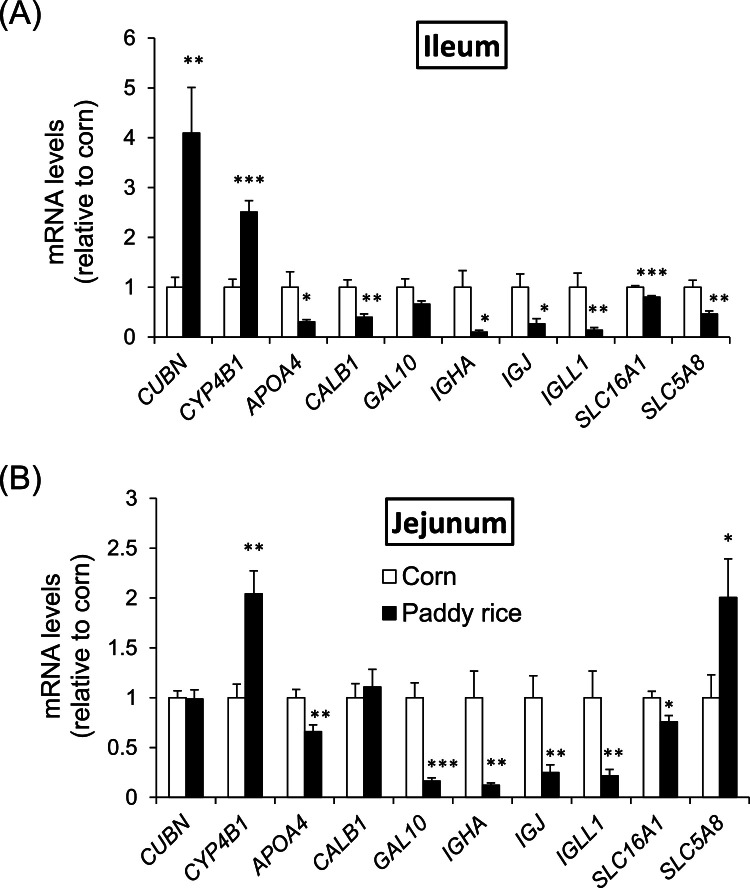
*CUBN, CYP4B1, APOA4, CALB1, GAL10, IGHA, IGJ, IGLL1, SLC16A1, and SLC5A8* mRNA levels in ileum (A) and jejunum (B) of chickens. Chickens were given corn or paddy rice-based diets from 7 to 21 days of age. Tissues were collected at 21 days of age, and mRNA expressions were assayed by real-time PCR. Vertical bars indicate means ± SEM, *n* = 9. *,**,***;Mean of paddy rice is significantly different from that of corn at *P* < 0.05(*), *P* < 0.01(**), and *P* < 0.001(***).

The expression levels of all genes were measured in the jejunum and ileum using real-time PCR to confirm the differences in gene expression. In addition to these genes, the expression of *IGHA*, the gene encoding the alpha chain of IgA, was measured because the probe of *IGHA* was lacking in the array used. The differences in gene expression levels in the ileum were consistent with the results of the DNA microarray analysis (Fig. 1a). Ileal *CUBN* and *CYP4B1* (*P* < 0.01) as well as *APOA4, CALB1, and IGHA* were upregulated. *IGJ, IGLL1, SLC16A1*, and *SLC5A8* were downregulated in paddy rice compared to in the control group (*P* < 0.05). Jejunal *IGHA, IGJ*, and *IGLL1* gene expressions were also downregulated in the paddy rice group (Fig. 1b).

### Experiment 2: serum and bile IgG and IgA concentrations and antibody titers against IBDV

**Fig. 2:**
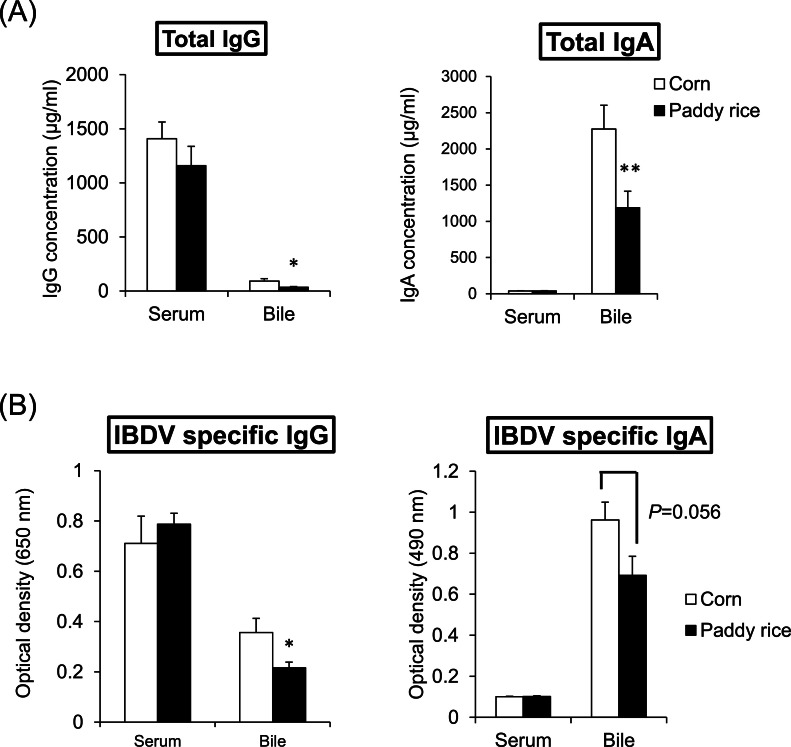
Antibody levels of chickens at 11 weeks (75 days) of age. Total IgG and IgA concentrations in serum and bile (A), and IBDV specific IgG and IgA levels in serum and bile (B). At 9 weeks of age, IBD vaccine was administrated to chickens by oral intubation and in drinking water. Vertical bars indicate means ± SEM, *n* = 7 (corn), 8 (paddy rice). Mean of paddy rice is significantly different from that of corn at *P* < 0.05(*) and *P* < 0.001(**).

Total and IBDV-specific IgG and IgA concentrations in the serum and bile were measured after oral exposure to a live IBD vaccine solution to investigate the effects of paddy rice consumption on antibody production in chickens. Total bile IgG and IgA concentrations were significantly lower in the paddy rice group than in the control group (*P* < 0.05), whereas serum total IgG and IgA concentrations did not change significantly (Fig. 2a). Similarly, while there were no differences in either IBDV-specific IgG or IgA levels in the serum between the two groups, while both IBDV-specific IgA levels and IgG levels in bile were 30–40 % lower in the paddy rice group (*P* < 0.056 for IgA and *P* < 0.05 for IgG; Fig. 2b).

### Experiment 3: SCFA concentrations in cecal contents

**Fig. 3:**
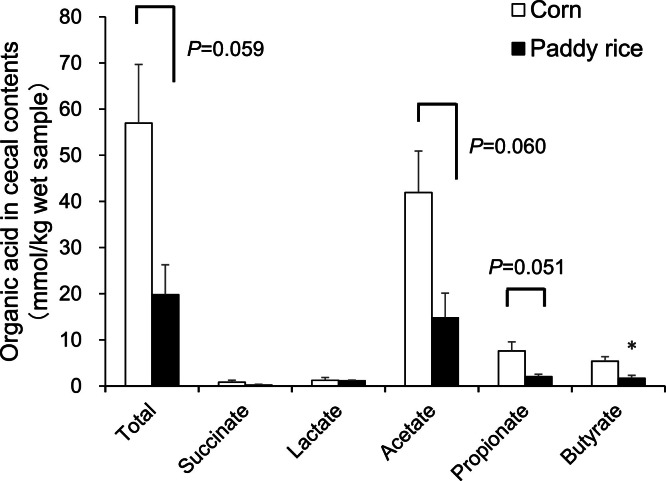
Short chain fatty acid concentrations in cecal contents. Chickens were given corn or paddy rice-based diets for 10 wks. Short chain fatty acid concentrations were measured by high performance liquid chromatography. Vertical bars indicate means ± SEM, *n* = 3. Mean of paddy rice is significantly different from that of corn at *P* < 0.05(*).

The production of SCFA in the cecal contents was determined to gain insight into how paddy rice ingestion reduces IgA production. Although there were no significant differences, the total SCFA, acetate, and propionate concentrations tended to decrease in the paddy rice group (*P* ≤ 0.06; Fig. 3). Only butyrate levels were significantly lower in the paddy rice group than in the control group (*P* < 0.05).

## Discussion

In the present study, paddy rice ingestion markedly decreased the intestinal gene expression of the immunoglobulin constituents *IGHA, IGJ*, and *IGLL1* compared to that of corn (Fig. 1). The bile total IgA and IgG concentrations and IBDV-specific IgA and IgG levels, which are indicators of gut immune responses in chickens (Murphy et al., 2022), were also lowered by paddy rice ingestion (Fig. 2), suggesting that paddy rice consumption weakened the gut immune response. However, neither blood IgA nor IgG concentrations differed between the corn and paddy rice groups. These results implied that the attenuated IgA and IgG production observed here is not systemic but rather a weakening of the antibody-producing response in the mucosal immune tissue of the intestinal tract.

Notably, paddy rice ingestion decreased the concentrations of SCFAs in the cecal contents compared with that under corn ingestion (Fig. 3). In support of this finding, the ileal gene expression of the SCFA transporters *SLC16A1* and *SLC5A8* was lowered by paddy rice consumption (Fig. 2). *SLC16A1* is a proton-conjugated transporter expressed on both the apical and basolateral sides of the intestinal epithelial cells, and its expression in the cecum and colon has been reported to increase with increasing fermentable fiber intake (Kirat et al., 2009). *SLC5A8* is a sodium ion-coupled transporter expressed on the apical side of the intestinal epithelial cells and has a higher affinity than *SLC16A1* (Gurav et al., 2015). Therefore, the low fermentability of paddy rice may have led to decreased expression of *SLC16A1* and *SLC5A8* in the intestine.

Recent studies have shown that SCFA delivery via a high-fiber diet promotes the host immunoglobulin response and plasma cell differentiation (Qu et al., 2023). The fermentability of fiber in the diet modulates immunoglobulin production, and its class switches to IgA in the gut (Nakajima et al., 2020). One possible mechanism is that SCFAs stimulate the energy production required for immunoglobulin production by B cells in gut-associated and secondary lymphoid tissues and promote differentiation into IgG- or IgA-producing plasma cells (Kim et al., 2016). In addition, acetic acid induces retinoic acid production in dendritic cells via the SCFA receptor GPR43 and promotes the IgA class switch in B cells (Wu et al., 2017). Moreover, Sanchez et al. (2020) confirmed that a modest concentration of SCFA promotes plasma cell differentiation via B cell-intrinsic epigenetic modulation. We speculated that the low fermentability of paddy rice leads to decreased IgA production in the intestinal tract. Paddy rice is rich in IDF compared to corn because of the presence of hulls. Acid detergent fiber, mainly cellulose and lignin, accounts for approximately 12.6 % of the dry weight in paddy rice and 2.9 % in corn (NARO, 2017). In addition, there is a difference in starch quality as a fermentation product between the paddy rice and corn. The starch can be classified as rapidly digestible, slowly digestible and resistant starch. Resistant starch has been defined as the fraction of starch which escapes digestion in the small intestine, and may be fermented as in the large intestine (Duan et al., 2024). Comparative study showed that resistant starch contents of the brown rice and corn were 1.5 % and 3.9 % dry matter basis, respectively (Deng et al., 2010). Paddy rice contains approximately 20-30 % of rice hull, so that the resistant starch content of paddy rice would be much less than that of the corn as well as brown rice. Therefore, the higher amount of nonfermentable fiber and the lower amount of resistant starch in paddy rice than in corn may have contributed to the lower fermentability of paddy rice, leading to poor antibody production.

Another explanation for the attenuated intestinal IgA and IgG production is that enhanced mucus secretion from paddy rice consumption protects the attachment or invasion of the IBDV vaccine strain in the gut. Our previous study suggested that feeding paddy rice increased the number of goblet cells and mucin secretion, resulting in protection against intestinal mucosal damage in DSS-induced colitis (Murai et al., 2018). The insoluble fibers that make up the hulls of paddy rice provide a physical stimulus to the intestinal tract and increase mucin secretion owing to their bulk-forming capacities (Tanabe et al., 2005). An increase in *MUC2* expression was observed following paddy rice ingestion in this study. This result raised the possibility that enhanced mucus secretion by paddy rice eliminated exposure to antigens/viruses, such that paddy rice ingestion might lower IBDV-specific IgA and IgG levels.

Based on microarray analysis, we also focused on genes involved in biological defense, nutrient utilization, and absorption in the gut. GAL10 is one of the antibacterial peptides, β-defensins. Antimicrobial peptides exert antimicrobial effects by altering the membrane permeability of the invading bacteria and damaging their metabolism. Previous studies have shown that mucins regulate the production of β-defensins and protect the commensal microbiota from the antimicrobial activity of β-defensins (Cobo et al., 2015). Hence, we hypothesized that the decrease in *GAL10* expression due to paddy rice consumption was due to increased mucin production. *CYP4B1* encodes a cytochrome P450 monooxygenase that catalyzes many endogenous reactions, including detoxification and activation (Ding et al., 2003). However, the function of *CYP4B1* in the small intestine of birds is unclear, and the cause of the increased expression of *CYP4B1* in the small intestine due to paddy rice consumption is unknown. The expression of *CUBN, APOA4*, and *CALB1*, which are involved in nutrient absorption, was altered by paddy rice consumption. *CUBN* encodes cubilin, a receptor for an endogenous factor and the vitamin B_12_ complex expressed in the ileum (Kozyraki, 2001). The intestinal microbiota influences the absorption of vitamin B_12_ in the intestinal tract (Wan et al., 2022). The consumption of paddy rice may have enhanced the expression of *CUBN* through modification of the microbiota. *APOA4* is an apolipoprotein that binds to lipids and transports them into the lymphatic fluid and blood circulation (Kohan et al., 2015). The crude fat contents of the feed sources used in this study were 3.22 % for corn and 1.46 % for paddy rice. Thus, it is likely that *APOA4* downregulation can be attributed to differences in the lipid content of the feed sources. In addition, studies using Caco2 cells have shown that butyrate enhances *APOA4* expression (Nazih et al., 2001). In the present study, paddy rice consumption decreased butyrate concentrations (Fig. 3), suggesting that a decrease in butyrate may attenuate *APOA4* expression. *CALB1* encodes calbindin, a high-affinity calcium-binding protein, whose expression in the intestine is modulated by vitamin D (Theofan et al., 1986; Bronner et al., 1986). As there was no difference in calcium content between the diets (corn diet: 1.09 %, paddy rice diet: 1.10 %), the cause of the altered *CALB1* gene expression is unknown. Overall, the present results and those of our previous study (Murai et al., 2018) confirmed that paddy rice consumption did not cause growth retardation, suggesting that the effects of paddy rice intake on nutrient absorption and availability were small.

In conclusion, the present results showed that paddy rice ingestion as a substitute for corn can alter a group of genes associated with immunity and gut barrier function, as well as the utilization of nutrients in the intestinal tract. This may be due to the physical properties of insoluble fibers abundant in paddy rice. In addition, paddy rice consumption reduced the intestinal immune response of chicks during oral vaccine exposure. However, it is still unclear whether the reduced immune response in the intestinal tract represents a weakening of anti-pathogenicity or a phenomenon caused by increased anti-pathogenicity via enhanced mucous secretion. Partially replacing corn with paddy rice, rather than replacing the entire amount of corn, can eliminate the relative risk of a reduced immune response.

## Declaration of competing interest

The authors declare that they have no known competing financial interests or personal relationships that could have appeared to influence the work reported in this paper.
